# Effect of fennel vaginal cream on sexual function in postmenopausal women: A double blind randomized controlled trial

**Published:** 2018

**Authors:** Parvin Abedi, Mahin Najafian, Masumeh Yaralizadeh, Foroogh Namjoyan

**Affiliations:** * PhD in Community Nutrition, Associate Professor in Midwifery Department, Menopause Andropause Research Center, Ahvaz Jundishapur University of Medical Sciences, Ahvaz, Iran; ** Department of Obstetrics and Gynecology, Imam Khomeini Hospital, Menopause Andropause Research Center, Ahvaz Jundishapur University of Medical Sciences, Ahvaz, Iran; *** MS.c in Midwifery, Midwifery Department, Menopause Andropause Research Center,Ahvaz Jundishapur University of Medical Sciences, Ahvaz, Iran; **** Department of Pharmacology, Marine Pharmaceutical Research Center, School of Pharmacy,Ahvaz Jundishapur University of Medical Sciences, Ahvaz, Iran

**Keywords:** Fennel vaginal cream, sexual function, sexual dysfunction, postmenopausal women

## Abstract

**Objective:** The aim of this study was to assess the effect of fennel on sexual function in postmenopausal women. It was a randomized controlled trial in 60 postmenopausal women with sexual dysfunction who were randomly assigned to two groups receiving either fennel vaginal cream (n=30) or placebo (n=30). Vaginal atrophy in the women was assessed using symptoms such as pallor, dryness, dyspareunia, itching and burning. The pH of the vagina and cytology of the vaginal mucosa were also measured at baseline and 8 weeks after the intervention. All participants were requested to fill out the Female Sexual Function Index (FSFI) at baseline and after 8 weeks. The intervention group was requested to use fennel vaginal cream (5 grams) every night, while the control group used placebo each night for 8 weeks. The data were analyzed using the independent t-test and Chi-square, Mann-Whitney and Wilcoxon tests. All areas of sexual function including arousal, lubrication, orgasm, sexual satisfaction and pain improved in both fennel and placebo groups after 8 weeks; however, the differences in the fennel group were more evident (p<0.05). The total FSFI score was significantly higher in the fennel group compared to the control group (8.2 ±9.4 and 8.03±10.36 before the intervention and changing to 33.79±0.7 and 18.99±1.09 after the intervention in the fennel and placebo groups, respectively; p<0.001).

**Discussion:** According to our results, fennel vaginal cream is an effective means of improving sexual activity in postmenopausal women. The use of this product in women who have sexual dysfunction and contraindications for hormone therapy is recommended.

## Introduction

Menopause is the permanent cessation of menstruation, and it is diagnosed by the lack of a menstrual period for at least 12 months [**1**]. Postmenopausal women experience physiological and psychological changes after menopause [**2**]. Some of the complications of menopause include hot flashes, insomnia, depression and dyspareunia [**3**]. Findings from the Global Study of Sexual Attitudes and Behaviors (GSSAB) involving 13,882 women in the 40-80 age range suggest that the prevalence of female sexual dysfunction (FSD) is 18 to 41%. The results of this study demonstrated that FSD may decrease the self-esteem and quality of life of women and may cause emotional distress [**4**]. A study conducted in Iran involving postmenopausal women showed that the prevalence of sexual dysfunction is 81.5% [**5**].

Estrogen is the most effective treatment for female sexual dysfunction that is approved by the US Food and Drug Administration (FDA) [**6**]. Estrogen given orally or vaginally can reduce vaginal dryness, vaginal atrophy and sexual dysfunction [**7**]. However, according to the results of the Women Health Initiative (WHI) study, hormone therapy did not improve general health, mental health, depression or sexual satisfaction among postmenopausal women [**8**]. The WHI study involving 16,608 postmenopausal women who were recruited to receive either estrogen and progesterone or placebo showed that after a 5.2-year follow-up, the risk of coronary heart disease, breast cancer, and endometrial cancer increased significantly among hormone users [**9**].

There are also natural treatments for sexual dysfunction. The results of some earlier studies showed that the herb Maca (Lepidiummeyenii) could significantly increase fertility and sexual activity in humans and animals [**10**]. In one study, niacin (1500 mg/day for 12 weeks) improved erectile problems in men compared to placebo [**11**]. Foeniculum vulgare or fennel is a plant in the carrot family and is indigenous to the shores of the Mediterranean Sea [**12**]. Iran is one of the producers of this plant. The main chemical compounds in fennel are trans-anethole and dianethole, which have estrogenic effects [**13**]. Using fennel significantly reduced hair thickness in women affected by hirsutism after 24 weeks [**14**].

The use of fennel for vaginal atrophy was assessed and approved for the first time in a recent study [**15**]. Also, the positive effect of fennel on sexual satisfaction in postmenopausal women has been reported in another study [**16**]. The purpose of the present study was to assess the effect of fennel on sexual function among postmenopausal women in Iran.

## Material and methods

This was a randomized controlled trial in which 60 postmenopausal women were recruited and randomly assigned to two groups: fennel and placebo (n=30 in each group). The design of this study was approved by the Ethics Committee of Ahvaz Jundishapur University of Medical Sciences, Ahvaz, Iran (Ref. No.: ajums.REC.1393.249). The protocol of this study was registered in the Iranian Randomized Controlled Trial Registry (Ref. No.: IRCT2014102919743N1). The inclusion criteria were as follows: women with age between 45 and 65 years, women who had natural menopause confirmed by amenorrhea for one year or increased follicle stimulation hormone (FSH)≥40 IU/l, women who lived with their husband and were sexually active, and score of Female Sexual Function Scale <26. Women with vaginal bleeding or infection, women who were receiving hormone therapy and women who used phytoestrogen, were excluded from the study.

All eligible women gave written informed consent before data collection. The following equation was used for sample size calculation [**17**]:

n=((s_1^2+s_2^2 ))/(x ®_1-x ®_2 )^2 〖(z_(1-α/2)+z_β)〗^2

In this formula, α = 0.05, β = 0.1, 1–β = 90%, P = 3.3%, p = 36%, and n = 26. The power of the study was set at 90%. We added 20% to the acquired number for attrition, and the total number of participants in each group was set at 30.

**Preparation of fennel and placebo**

Both fennel and placebo were made in the Pharmacology School Laboratory of Ahvaz Jundishapur University of Medical Sciences. Fennel seeds were mixed with ethanol 80% and stored for three days. The mixture was then dried using a rotary and freezer dryer. The dried fennel extract was mixed with 5% emulsion cream base. For preservation, propylparaben and methylparaben were added to oil and water phases, respectively. The fennel extract was mixed with a proper carrier and prepared with a concentration of 5% [**18**]. The placebo cream was manufactured using the proper carrier with a similar color and appearance to the fennel cream. Details of the preparation of fennel and placebo have been published previously [**15**].

**Randomization**

Eligible women were randomly assigned into one of the two groups: fennel or placebo. Fennel vaginal cream or placebo was coded as “A” or “B” by a person who was not aware of the purpose of the study. One of the researchers (MY), who was not aware of the encoded fennel or placebo, was responsible for distributing the fennel cream or placebo to the women.

**Measures**

A demographic questionnaire and the Female Sexual Function Index (FSFI) were used to collect data. The FSFI contains 19 questions in six domains of the sexual function. In this questionnaire, two questions were dedicated to measuring sexual desire, four for measuring arousal, four for measuring lubrication, and three for measuring each orgasm, satisfaction and pain. The score of each domain was multiplied by a special factor; 0.6 for desire, 0.3 for arousal and lubrication and 0.4 for other areas of sexual function. The minimum and maximum scores for all domains are 2 and 36, respectively. A score less than 26 indicates sexual dysfunction. The validity and reliability of this questionnaire have been tested by Rosen et al. [**19**]. The reliability and validity of the Persian version of this questionnaire were evaluated in Iran by Fakhri et al., and the results showed that this questionnaire has acceptable validity and reliability for use in Iranian women [**20**]. All women were requested to complete the FSFI questionnaire at baseline and 8 weeks after the intervention, while one of the researchers (MY) was present to clarify any ambiguity for the participants.

Postmenopausal women were also assessed regarding subjective and objective signs and symptoms of vaginal atrophy. The results of vaginal atrophy assessment were previously published [**15**].

**Statistics**

All data were entered into Statistical Package for the Social Sciences (SPSS), version 22. Continuous data were tested for normality using the Kolmogorov-Smirnov test. The independent t-test and Mann-Whitney U test were used to compare the two groups regarding continuous data. The Wilcoxon signed-ranked test was used to compare sexual function before and after the intervention. The chi-square test was used to compare categorical data between the two groups.

## Results

**Fig. 1:**
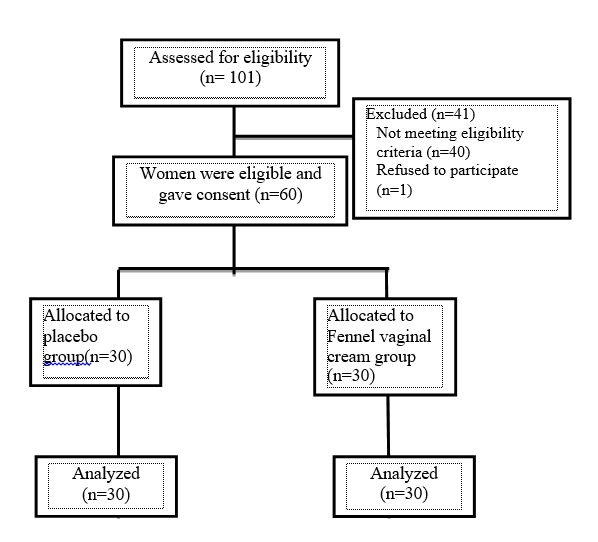
The flow diagram of recruitment and retention of participants in the study

In this study, we did not have any drop-outs. **Figure 1** indicates the recruitment and retention of the participants. The mean age of women in the fennel group was 53.73±3.6 and 52.9±3.4 years in the control group (p>0.05). The two groups did not show any significant difference regarding the age of menopause, body mass index, education or economic situation. Most women in the two groups had one coitus per week (43.3% vs. 40% in the fennel and control groups, respectively; p>0.05) (**Table 1**).

**Table 1:** The socio-demographic characteristics of participants in the Fennel and placebo groups

Variables	Fennel group n=30	Placebo group n=30	P value
	Mean±SD or N(%)		
Age (y)	53.73±3.6	52.9±3.4	0.36
Age of menopause (y)	49.5±1.99	49.3±1.9	0.32
Body mass index (kg/m2)	24.6±3.9	23.4±3.2	0.20
Education			
High school	20(66.7)	16(53.3)	0.50
Diploma	7(23.3)	8(26.7)	
University education	3910)	6(20)	
Job			
Employee	5(16.7)	4(13.3)	0.59
House keeper	23(76.7)	21(70)	
Retired	2(6.7)	5(16.7)	
Economic situation			
Weak	9(30)	11(36.70	0.83
Moderate	14(46.70	12(40)	
Well off	7(23.3)	7923.30	
Numbers of coitus per week			
0	2(6.7)	3(10)	0.94
1	13(43.3)	12(40)	
2	6(20)	7(23.3)	
3	7(23.3)	5(16.7)	
4	4(6.7)	3(10)	

**Table 2** gives the mean score of the sexual function domain in the fennel and control groups. This table shows that desire increased in the fennel and control groups significantly after 8 weeks (from 2 ± 0.53 to 5.3±.36 in the fennel group, p<0.001; from 1.8±0.6 to 2.9±0.46 in the control group, p<0.001). Desire increased in the fennel group more than it did in the control group (p<0.001). Other domains of sexual function increased significantly in each group after 8 weeks; however, the fennel group showed significant improvement in all areas (p<0.001).

**Table 2:** The mean score of the sexual function domain before and after the intervention in the two groups

Variables	Fennel (N=30)		Control (N=30)		P value between groups
	Before	After	Before	After	
	Mean ± SD		Mean ± SD		
Desire	2 ± 0.53	5.3±0.36*	1.8±0.6	2.9±0.46*	<0.001
Arousal	1.13±0.56	5.3±0.36*	1.13±0.66	2±0.32*	<0.001
Lubrication	1.5±0.89	5.7±0.27*	3.6±0.86	3.3±0.38*	<0.001
Orgasm	1.7±0.88	5.6±0.28*	1.8±1.09	3.4±0.48*	<0.001
Sexual satisfaction	1.36±0.36	5.8±0.26*	1.28± 0.28	3.5±0.41*	<0.001
Pain	1.45±0.78	5.8±0.24*	1.32±0.91	3.3±0.26*	<0.001
Total score of sexual function	9.4±2.8	33.79±0.78*	10.36±8.03	18.9±1.09*	<0.001


*P<0.001 Using Wilcoxon Signed Ranks Test

The total FSFI score was significantly higher in the fennel group compared to the control group after the intervention (8.2 ±9.4 and 8.03±10.36 before intervention and 33.79±0.7 and 18.99±1.09 after the intervention in the fennel and placebo groups, respectively; p<0.001). None of the intervention or control groups reported any side effects from fennel or placebo.

## Discussion

This study aimed to assess the effect of fennel on sexual function in postmenopausal women. Our results showed that all areas of sexual function significantly improved in the fennel group compared to the control group. Foeniculum vulgare or fennel is a plant that has estrogenic effects. This plant can improve milk secretion, promote menstruation, and decrease postmenopausal symptoms in women and andropause symptoms in men [**21**]. The positive effect of fennel on the vaginal and cervical epithelium of rats has been reported [**18**].

Jaroenporn et al. conducted a study on 12 postmenopausal cynomolgus macaques with cessation of menstruation for at least 5 years, to compare the effect of a vaginal cream with Puerariamirifica extract and a conjugated equine estrogen cream. Their results showed that this vaginal cream significantly improved vaginal atrophy (an increase in superficial cells and a decrease in the pH of the vagina) similar to conjugated equine estrogen, without any side effects [**22**]. These results in terms of improving vaginal atrophy are similar to what we found in our study with fennel vaginal cream.

A study conducted by Yaralizadeh et al. that evaluated the effect of fennel on vaginal atrophy in postmenopausal women also showed that fennel could significantly increase the number of superficial cells of the vagina, decrease the pH and the objective and subjective symptoms of vaginal atrophy (pallor, itching, dryness and dyspareunia) [**15**]. In another part of the aforementioned study, the effect of fennel on sexual satisfaction in 60 postmenopausal women was assessed. The results showed that fennel significantly increased sexual satisfaction in postmenopausal women compared to placebo [**16**]. The results of the present study are in line with the two studies mentioned above, as all of them demonstrated that fennel could significantly decrease vaginal atrophy and improve sexual satisfaction and also sexual function.

A study conducted by Abdali et al. compared an oral tablet of fennel with Hypericumperforatum regarding their effect on climacteric symptoms and sexual activity in 120 postmenopausal women aged 45-60 years. The results of this study showed that the total score of sexual function in both fennel and Hypericum groups increased significantly compared to the placebo group (increased from 18.2±2.61 to 22.78± 2.61 in the Hypericum group, from 18.38±2.29 to 22.51±2.25 in the fennel group and from 19.39±3.18 to 20.79±3 in the placebo group; p<0.01) [23]. These results are similar to what we found in our study. However, there is a difference between these two studies as Abdali et al. used an oral form of fennel and we used a vaginal cream, and furthermore, they only assessed the total score of sexual function, while we assessed different domains of sexual activity plus the total score of sexual activity. Also, the total score of sexual function in our study improved better than it did in Abdali et al.’s study.

**Strength and limitations of study**

Fennel vaginal cream was introduced in the first part of this study [**15**] and patented in Iran (Ref. No.: 034356). Also, a patent application has been filled for it at the United States Department of Commerce (United States Patent and Trademark Office) (Confirmation No.: 5013).

One of the limitations of this study was that talking about sexual matters in the Iranian culture is not simple. Participants may not express their true feelings about sexuality. We only relied on participants’ responses to the questions in the FSFI. We only followed participants for 8 weeks. Perhaps with a longer follow-up, the advantages and disadvantages of the fennel vaginal cream could be better assessed.

## Conclusion

According to our results, fennel vaginal cream is an effective means to improve the sexual activity of postmenopausal women. The use of this product in women who have sexual dysfunction and contraindications for hormone therapy is recommended.

**Acknowledgment**

This study was part of the master thesis of MY. All expenses of this research were provided by Ahvaz Jundishapur University of Medical Sciences. We would like to thank all postmenopausal women who participated in this study.

**Source of funding:** Ahvaz Jundishapur University of Medical Sciences provided all expenses of this research.

**Disclosures:** The authors declare that there is no conflict of interest.
